# Supplementation of *Acacia dealbata* versus *Acacia mearnsii* leaf-meal has potential to maintain growth performance of lambs grazing low-quality communal rangelands in South Africa

**DOI:** 10.1007/s11250-024-04004-z

**Published:** 2024-05-09

**Authors:** L. H. Mushunje, T. Marandure, O. C. Chikwanha, J. Bennett, H. J. Hawkins, A. R. Palmer, L. Wu, Marufu M.C, C. Mapiye

**Affiliations:** 1 Department of Animal Sciences, Stellenbosch University, P. Bag X1, Matieland, 7602 South Africa; 2 Centre for Agroecology, Water and Resilience (CAWR), Coventry University, Wolston Lane, Ryton Gardens, Coventry, CV8 3LG UK; 3 Department of Energy and Technology, Swedish University of Agricultural Sciences, Box 7032, Uppsala, SE-75007 Sweden; 4 Conservation South Africa, Forrest House, Belmont Park, Rondebosch, 7700 South Africa; 5 Department of Biological Sciences, University of Cape Town, Private Bag X1, Rondebosch, 7701 South Africa; 6 Institute for Water Research, Rhodes University, PO Box 94, Makhanda, 6140 South Africa; 7 Key Laboratory of Aquatic Botany and Watershed Ecology, Wuhan Botanical Garden, Chinese Academy of Sciences, Wuhan, 430074 China; 8 Department of Veterinary Tropical Diseases, Faculty of Veterinary Science, University of Pretoria, Onderstepoort, 0110 South Africa

**Keywords:** *Acacia* foliage, Faecal egg count, Growth performance, Polyphenols, South Africa

## Abstract

Supplementing livestock grazing communal rangelands with leaf-meals from *Acacia* trees, which are currently considered as problematic invasive alien plants globally, may be a sustainable way of exploiting their desirable nutritional and anthelmintic properties. The current study evaluated worm burdens and growth performance of lambs grazing low-quality communal rangelands supplemented with leaf-meals prepared from the invasive alien plant species; *Acacia mearnsii* or *A. dealbata*. Forty, three-month-old ewe lambs weighing an average of 18.9 ± 0.60 kg were randomly allocated to four supplementary diets: (1) rangeland hay only (control), (2) commercial protein supplement plus rangeland hay, (3) *A. mearnsii* leaf-meal plus rangeland hay and (4) *A. dealbata* leaf-meal plus rangeland hay. All the supplementary diets were formulated to meet the lambs’ minimum maintenance requirements for protein. All the lambs were grazed on communal rangelands daily from 0800 to 1400 after which they were penned to allow them access to their respective supplementary diets until 08:00 the following morning. The respective supplementary diets were offered at the rate of 400 g ewe^− 1^ day^− 1^ for 60 days. Lambs fed the commercial protein supplement had the highest dry matter intake followed by those fed the *Acacia* leaf-meals and the control diet, respectively (*P* ≤ 0.05). Relative to the other supplementary diets, lambs fed the commercial protein supplement and *A. dealbata* leaf-meal had higher (*P* ≤ 0.05) final body weight and average daily gains. Dietary supplementation did not affect lamb faecal worm egg counts over the study period (*P* > 0.05). There was no association between supplementary diets and lamb FAMACHA© scores (*P* > 0.05). It was concluded that supplementation of *Acacia dealbata* versus *Acacia mearnsii* has the potential to emulate commercial protein in maintaining growth performance of lambs grazing communal rangelands in the dry season.

## Introduction

Seasonal fluctuations in quality and quantity of natural forage are largely responsible for the inferior performance of livestock grazing communal rangelands in the tropics (Mphinyane et al., 2015; Mthi and Nyangiwe, 2018). A significant drop in nutritive value, particularly crude protein (CP) from about 12% in the wet season to as low as < 2% in the dry season (Mapiye et al., 2009; Moyo et al., 2012) result in drastic shortfalls in meeting the protein requirements for both maintenance and production of sheep (NRC, 2007). In addition, the proliferation of invasive alien plants (IAPs), especially Australian Acacias (i.e., *Acacia dealbata* and *A. mearnsii*) in Africa, especially in South Africa has reduced rangeland production (Yapi et al., 2018; Gouws and Shackleton, 2019) and impacted negatively on livestock health, meat production and quality (Dezah et al., 2021; Uushona et al., 2021). In South Africa, invasive Acacias are currently harvested by local communities for timber and fuelwood (Ngorima, 2014) and this often yields by-products such as leaves and barks, which currently have limited local value. Strategies are being developed in the tropics to increase the economic value of invasive Acacias’ leaves and barks to local communities, including their use as livestock feed and meat preservatives because they contain moderate to high levels of nutrients and polyphenols (Dezah et al., 2021; Uushona et al., 2021; 2022).

Effective exploitation of leaves from *Acacia* species could be a sustainable way of managing invasions by these alien plants and may provide a lucrative alternative to more expensive processed protein sources for resource-poor farmers, whose animals mostly rely on communal rangelands throughout the year (Mapiliyao, 2012; Maguraushe, 2015, Mbambalala, et al., 2023). Communal farmers have previously attested to observing increased browsing of Australian Acacias leaves by ruminants in the dry season to compensate for the decline in forage quality (Mphinyane et al., 2015; Mushunje, 2022). Dezah et al. (2021) and Uushona et al. (2021) reported consistently high levels of CP (16.2%) and NDF (36.1%) in Australian *Acacia* foliage. This is consonant with Acacias being N-fixing and staying green during the dry season due to their long tap roots that can reach deep, ground water sources (Mapiliyao, 2010; Maguraushe, 2015; Mbambalala et al., 2023). However, high contents of condensed tannins (CT) in *Acacia* leaves, adversely affects their intake and digestibility by ruminants (Payne et al., 2013; Chikwanha et al., 2023; Uushona et al., 2023). Co-feeding these browse species with low condensed tannin-containing ingredients such as rangeland hay could be a cost-effective way of reducing the negative effects of secondary metabolites (Salem and Smith, 2008).

Apart from being limited by low quantity and quality forages, particularly in the dry season, ruminant livestock production on communal rangelands is compromised by high loads of gastrointestinal parasites (Mapiliyao, 2010, Mushunje, 2022). Given the predominance of sheep over other ruminant livestock among communal farmers in the Eastern Cape Province of South Africa (StatsSA, 2017), it is important to focus on the species for testing the complementary anthelminthic and feed supplement potential of acacia leaf-meals. Previous studies that investigated the feed and anthelminthic potential of Acacia species on livestock growth performance and parasitic worm burden largely focused on bark tannin extracts. For example, Cenci et al. (2007), observed better growth performance and lower nematode loads in sheep fed *A. mearnsii* extracts containing up to 3.2 gCT kg^− 1^ DM. On the contrary, low average daily gain (ADG) and carcass weights were reported for lambs fed *A. mearnsii* bark extracts containing ≥ 60 g CT kg^− 1^ DM (Costa et al., 2021). Recently, feeding *A. mearnsii* leaf-meal up to 100 gkg^− 1^ DM replacing *Triticum aestivum* (wheat) bran in lamb finisher diets had no effect on growth performance and carcass weights (Uushona et al., 2023b). To the authors’ knowledge, there are few if any studies that assessed the use of *Acacia* leaf-meals to supplement sheep grazing tropical communal rangelands in the dry season. The objective of the current study was, therefore, to evaluate the effects of supplementing *A. mearnsii* and *A. dealbata* leaf-meals on worm burdens and growth performance of lambs grazing South African communal rangelands in the dry season.

## Materials and methods

### Study area

The study was conducted in the Magxeni community in the Mvenyane area (Matatiele, Eastern Cape, South Africa; (30° 32’ 13” S 29° 01’ 32"E). The community was purposively selected because of its active involvement in programs initiated by a non-profit organization (NPO) called Conservation South Africa (CSA). The NPO assisted farmers in the Magxeni community to form a grazing association to help them practice sustainable rangeland and livestock management in return for various incentives, including livestock vaccinations, organising sheep auctions and offering farmer training programs. Magxeni community lies within the East Griqualand Grassland vegetation unit of the sub-escarpment Grassland bioregion (Mucina and Rutherford, 2006). It is characterised by well-drained, erosion-prone soils and hilly terrain with an altitude range of 920–1740 m above sea level. The vegetation in the study area’s rangelands comprises of mix of grass species dominated by *Themeda triandra* with *Tristachya leucothrix, Heteropogon contortus, Eragrostis curvula* and *Andropogon appendiculatus* also available (Mucina and Rutherford, 2006). The region receives summer rainfall and winter snowfall (at high altitudes), with about 30 days of annual frost, mean annual precipitation of approximately 780 mm and a mean annual temperature of 14.7 °C (Gouws and Shackleton, 2019). The area is heavily invaded by *Acacia* species up to 7000 stems/ ha with an annual increase of the *Acacia* biomass invasion estimated to be 26% (Gouws and Shackleton, 2019). In Mvenyani district about 50% of the area is invaded by *A. mearnsii* and *A. dealbata* (Gouws and Shackleton, 2019).

### Sourcing of feed ingredients and preparations of experimental diets

*Acacia* leaf-meals were harvested within a 10 km radius of the study site between September and October 2020 by cutting small *A. mearnsii* and *A. dealbata* trees (1.5–2.0 m tall) with a hand-held petrol chainsaw. Trees were cut into small branches, which were then stacked on plastic sheets and sun-dried for four days. On the fifth day, dry leaves were shaken off branches, collected from the plastic sheet, bagged and stored in a well-ventilated, dry shaded area. Rangeland hay (∼ 70 g CP/ kg DM) harvested in the same season was sourced from a nearby commercial farm and ground using a hammer mill fitted with a 30 mm sieve to allow mixing with the *Acacia* leaf meals. A commercial protein supplement (Protein Lick 40, Molatek, South Africa) was purchased from an animal feed store in Matatiele (Eastern Cape Province, South Africa).

Supplementary diets were formulated using a Pearson square method to meet the lambs’ daily minimum maintenance requirements for protein (i.e., 70 g CP/ kg DM; NRC, 2007). The diets were prepared by mixing rangeland hay with either commercial protein or *Acacia* leaf-meals supplements. The four diets formulated contained (1) rangeland hay only, (2) 94% rangeland hay plus 6% commercial protein supplement, (3) 78% rangeland hay plus 22% of either *A. mearnsii* leaf-meal and (4) or *A. dealbata* leaf-meal. The supplementary diets were bagged, labelled, and stored in a well-ventilated cool-dry shed before feeding. All the diets were premixed at the beginning of the trial and safely stored in a dry, well-ventilated place for the duration of the trial.

### Nutritional composition of experimental diets

Samples from rangeland biomass and each diet and were collected weekly and separately pooled for analyses of nutritional composition. Feed ingredients and diets were analysed for dry matter (DM), ash and ether extract (EE) using AOAC International (2002) procedures. A macro-Nitrogen analyser (LECO FP528, LECO Corporation, Miami, USA) was used to determine the total nitrogen content which was multiplied by a factor of 6.25 to calculate crude protein (CP) content. Neutral detergent fibre (NDFom), acid detergent fibre (ADFom) and acid detergent lignin (ADL) of the feed ingredients and diets were analysed using an ANKOM 200 Fibre Analyzer (Ankom Technology Corp., Macedon, NY, USA). Total phenol and tannins were determined using Makkar’s (2003) method. Condensed tannins were determined following the procedure by Porter et al. (1985). Analyses of all the proximate, fibre and polyphenolic compositions were done in triplicate. The nutritional composition of the experimental diets and rangeland biomass are shown in Table 1.

**Table 1:** Nutritional composition of the experimental diets and rangeland biomass (%)

Parameter	Diets					Rangeland biomass			
	Rangeland hay (control)	Protein supplement	A. mearnsii	A. dealbata		August	September	October	SD
Dry matter (%)	79.9	79.9	82.7	72.7		85.7	75.3	83.9	4.30
Crude protein (%)	6.9	9.8	8.1	8.6		7.2	6.4	8.6	0.14
Ether extract (%)	0.8	0.9	0.8	0.6		0.7	0.4	0.4	0.07
Ash (%)	4.2	10.1	4.2	4.6		5.2	4.8	5.1	0.11
Neutral detergent fibre (%)	67.6	65.0	62.3	61.5		66.0	68.2	66.1	1.87
Acid detergent fibre (%)	37.4	36.4	35.6	34.9		37.2	36.8	33.7	0.40
Acid detergent lignin (%)	37.9	36.6	35.9	35.2		37.2	37.2	34.2	0.41
Metabolisable energy (MJ/kg DM)	7.7	7.9	8.1	8.3		7.7	7.8	8.6	0.34
Total phenolics (g /kg DM)	NA	NA	23.0	20.1		NA	NA	NA	0.10
Total tannins (g/kg DM)	NA	NA	2.2	2.6		NA	NA	NA	02.8
Condensed tannins (g/kg DM)	NA	NA	10.3	14.3		NA	NA	NA	1.16

SD – standard deviation, DM – dry matter, NA – not applicable

### Management of experimental animals

Ethical approval was obtained from the Stellenbosch University Research Ethics Committee for Animal Care and Use (ACU-2020‐11,758). Forty, 3-months-old Dohne Merino ewe lambs (18.9 ± 0.60 kg) were purchased from a commercial farmer in Matatiele. The animals were ear-tagged for identification but not drenched for internal parasites. Neither were tests for the presence of gastrointestinal parasites conducted on the lambs prior to the study. Given the high prevalence of gastrointestinal parasites in the study area (Mushunje, 2022) and management of the study lambs as a single cohort from lambing, they were assumed to have similar loads of gastrointestinal parasites.

Ewe lambs were randomly allocated to the four treatment diets. Each day the lambs grazed communal rangelands and drank water from the river between 0800 and 1400. They were expected to consume 60% of their daily feed intake within the 6 h grazing period Mohammed et al., 2020). For the remainder of each day, the animals were individually housed in pens measuring 1.5 × 1.5 m from 1400 to 0800 and provided with supplementary diets at the rate of 400 g ewe^− 1^ day^− 1^ (∼ 2% of body weight) and offered clean freshwater *ad libitum.* The animals were adapted to the supplementary diets for 21 days followed by 60 days of data collection (August to October 2021).

### Feed intake and body weights of lambs

During the trial, all the feed offered, and refusals were weighed and recorded daily before feeding in the morning. Feed was given at 10% extra from the previous day’s consumption. Lambs were weighed at the beginning of the experiment and thereafter biweekly to give a total of seven measurements until the end of the experimental period using a commercial scale (Rudd scale, Durbanville, South Africa). The animals were weighed in the morning (0600) before being released for grazing. The average daily weight gain was calculated by subtracting the initial body weight from the final body weight and divided by the number of days on feed excluding the adaptation period.

### Faecal worm egg counts and FAMACHA scores

Faecal samples were collected directly from the rectum using the faecal grab technique (Da Costa et al., 2019). Samples were placed in labelled zip lock bags, temporarily stored in a cooler box at 4 °C and transported to Queenstown Provincial Veterinary laboratories for determination of faecal nematode egg counts using the McMaster Technique (Miller, 1997). The number of nematode eggs of the McMaster chamber was multiplied by 50 to obtain eggs per gram. FAMACHA scores were determined every two weeks during the trial. Each animal was visually examined by a trained person and classified into one of five categories according to the FAMACHA© eye colour chart (Van Wyk, 2008).

### Statistical analyses

All the data were analysed with SAS v. 9.4 (SAS Institute Inc. Cary, NC, USA). Before analysis, the faecal worm egg count (FEC) values were checked for normality using the PROC UNIVARIATE procedure and transformed to conform to normality by a box-cox transformation using the following formula; $y\lambda =\frac{{y}^{\lambda }-1}{\lambda }$, (λ = 0.25), where *y* is the response variable and λ is estimated using the maximum likelihood estimate adapted from the algorithm proposed by Hyde (1999) using the TRANSREG procedure SAS v. 9.4 (SAS Institute Inc. Cary, NC, USA).

The effect of diet on growth performance and FEC was analysed using PROC MIXED procedures SAS v. 9.4 (SAS Institute Inc. Cary, NC, USA). Diet, time on feed and their interaction were fitted as fixed effects, while the animal within a diet was fitted as a random variable, time on feed was a repeated measure and initial weight was a covariate. The following model was used: Y_*ijk*_ = µ + D_*i*_ + T_*j*_ + (DT)_*ij*_ + ε_*ijk*_; Where: Y_ijk_ = final weight, ADG, faecal egg count; µ = overall mean; D_i_ = effect of diet (i = *A. mearnsii*, *A. dealbata*, protein supplement and rangeland hay only); T_j =_ Time on feed (j = week 0, 2, 4, 6 and 8); (DT)_ij_ = interaction _i_^th^ diet and _j_^th^ time on feed and ε_ijk_ = residual error. Pair-wise comparisons of the least square means were performed using the PDIFF test with a TUKEY adjustment in SAS version 9.4. The significance threshold for all statistical analyses was set at *P* ≤ 0.05. The association between diet and FAMACHA© scores was performed using the chi-square test.

## Results

### Nutritional composition of the experimental diets and rangeland biomass

The nutritional composition of the experimental diets and the rangelands are shown in Table 1. The protein supplement diet had greatest CP content followed by the *Acacia* leaf-meal diets with the rangeland hay diet (control) having the least. Relative to the other diets, ash content was highest for the protein supplement diet. The fibre components (NDF, ADF and ADL) decreased in the order of rangeland hay > protein supplement > *A. mearnsii* > *A. dealbata*. The *Acacia dealbata* had higher CT than *A. mearnsii.* Rangeland grazing hay for October had higher CP content and lower ADF and ADL contents than the other months.

### Effect of diet on growth performance of lambs

**Fig. 1:**
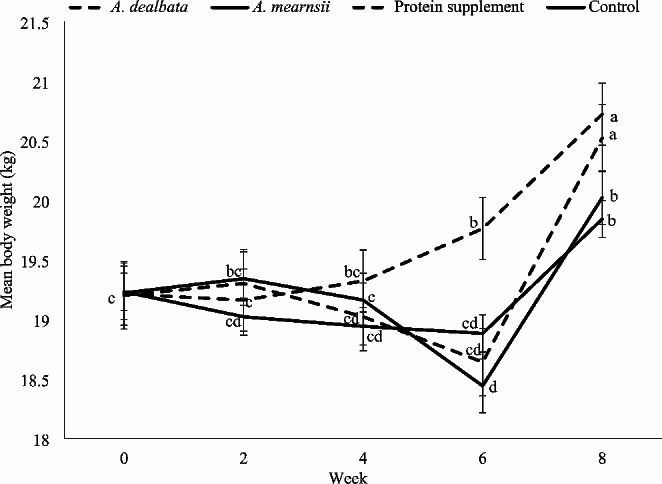
Least square means (± standard error of mean) of body weights of lambs grazing South African communal rangelands supplemented with Acacia leaf-meals. ^*a−b*^ Values with different superscripts are significantly different (*P* ≤ 0.05)

The commercial protein supplement diet had the highest DMI with intermediate values observed for the *Acacia* diets and the control diet had the least intake (*P* ≤ 0.05; Table 2). Diet and time on feed interaction influenced weight of lambs (*P* ≤ 0.05; Fig. 1). Body weight increased for lambs on the *A. dealbata* diet until week 2 thereafter declined until week 6 and then increased to week 8 (*P* ≤ 0.05). Body weight of lambs on *A. mearnsii* gradually declined until week 6 and then increased up to week 8 (*P* ≤ 0.05). Lambs on the protein supplement consistently increased (*P* ≤ 0.05) weights throughout the trial period. Body weights for control lambs increased to week 2 and thereafter declined to week 6 and then increased to week 8 (*P* ≤ 0.05). Lambs fed the protein supplement and *A. dealbata* diets had similar (*P* > 0.05) final body weight and ADG, which were higher (*P* ≤ 0.05) than that the control and *A. mearnsii* dietary treatments, which were not different (*P* > 0.05) from each other.

**Table 2:** Least square means (± standard error of mean) of growth performance of lambs grazing South African communal rangelands supplemented with Acacia leaf-meals

Parameter	Diet				*P*-value
	Rangeland hay	Protein supplement	A. mearnsii	A. dealbata	
Initial body weight (kg)	19.2 ± 0.06	19.2 ± 0.06	19.2 ± 0.05	19.2 ± 0.06	0.2532
Final body weight (kg)	20.0 ± 0.01^b^	20.7 ± 0.03^a^	19.8 ± 0.04^b^	20.5 ± 0.02^a^	0.0499
Dry matter intake (g)	274.4 ± 5.22^c^	318.9 ± 4.92^a^	288.6 ± 5.01^b^	290.4 ± 5.21^b^	< 0.0001
Average daily gain (g)	13.7 ± 2.67^b^	25.0 ± 3.07^a^	10.7 ± 2.33^b^	21.9 ± 3.67^a^	0.0399

^*a, b*^ Values with different superscripts within a row are significantly different (*P* ≤ 0.05)

### Effect of diet on faecal worm egg counts and FAMACHA scores

**Fig. 2:**
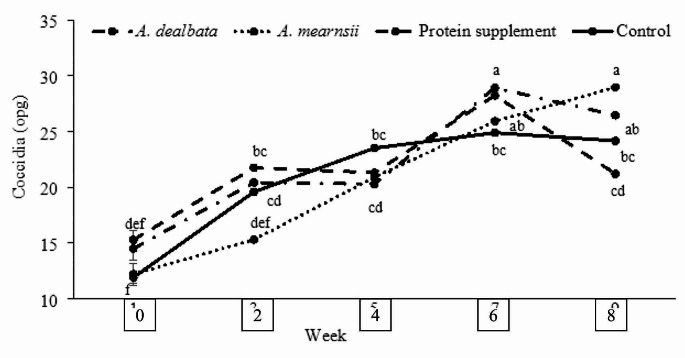
Least square means (± standard error of mean) of coccidia egg counts in lambs grazing South African communal rangelands supplemented with Acacia leaf-meals. ^a−*f*^ Values with different superscripts are significantly different (*P* ≤ 0.05)

**Fig. 3:**
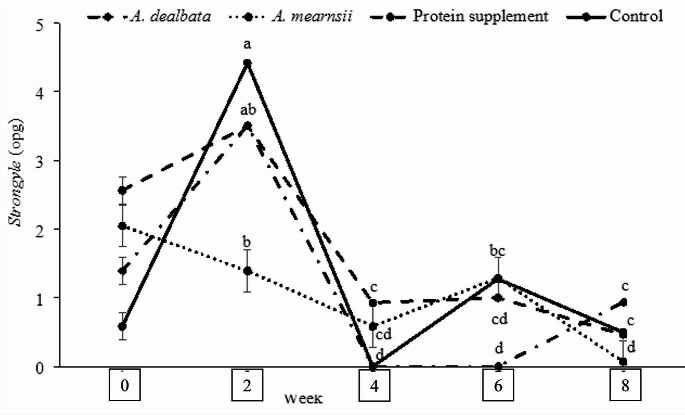
Least square means (± standard error of mean) of strongyles in lambs grazing South African communal rangelands supplemented with Acacia leaf-meals. ^*a−e*^ Values with different superscripts are significantly different (*P* ≤ 0.05)

Diet and time on feed interaction influenced coccidia and strongyle egg counts (*P* ≤ 0.05; Fig. 2). Faecal egg counts for coccidia increased for all the diets from the beginning of the trial to week 6 and then declined to week 8 except for the *A. mearnsii* diet FEC which increased throughout the trial (*P* ≤ 0.05). At the end of the trial, coccidia FEC were in the order of *A. mearnsii* > *A. dealbata* > rangeland hay > protein supplement (*P* ≤ 0.05). For the *A. mearnsii* diet, strongyles FEC declined from beginning until week 4 and plateaued to week 8, while the FEC for the rest of the diets increased from the beginning to week 2, declined (to week 4 and then remained constant until week 8 without significant differences among them (*P* > 0.05; Fig. 3). There was no association between supplementary diet and FAMACHA scores (*P* > 0.05; Table 3). However, at the end of the trial, there were more lambs observed to be mildly anaemic (C3; Table 3) than at the beginning of the experiment.

**Table 3:** FAMACHA scores (%) of lambs grazing South African communal rangelands supplemented with Acacia leaf-meals

Parameter	Control	Protein supplement	A. mearnsii	A. dealbata	*P*-values	Cramer’s V
*Initial scores*						
Non-anaemic (red ocular membrane, A1)	7.5	10	10	10	0.8741	0.1208
Non-anaemic (red/pinkish ocular membrane, B2)	17.5	15	15	15		
*Final scores*						
Non-anaemic (red/pinkish ocular membrane, B2)	2.8	0	5.6	2.8	0.4517	0.2826
Mildly anaemic (pink ocular membrane, C3)	19.4	22.2	16.7	22.2		
Anaemic (pink, white ocular membrane, D4)	2.8	5.6	0	0		

## Discussion

The lower average DMI recorded for the rangeland hay and *Acacia* diets compared to the protein supplement diet could be attributed to palatability of the respective diets. It has been demonstrated that palatability is positively correlated with solubility of the dietary components, with protein diets being more soluble, whereas structural components, such as NDF, ADL and phenols present in mature hay and *Acacia* diets respectively are less soluble (Rubanza et al., 2007; Gebremeskel et al., 2019). A combination of low CP and high fibre (i.e., NDF and ADL) as well as phenols negatively influenced DMI of the test diets compared to the control. The relatively high levels of CT present in the *Acacia* diets negatively influenced palatability through the formation of a tannin-salivary protein complex that causes an astringent taste in the mouth by producing a feeling of constriction, dryness and roughness in the oral cavity (Waghorn, 2008; Jerónimo et al., 2016; Chikwanha, 2023). Moreover, CT inhibit activities of ruminal microorganisms by binding to proteins and forming stable complexes in the rumen (Brown et al., 2018; Priolo et al., 2000). Priolo et al. (2000) reported that a diet containing 2.5% CT reduced feed palatability and digestibility. Although CT levels in the Acacia diets used in the current study are well below 2.5%, their combination with fibre and other phenols could have contributed to the reported differences in DMI across the experimental diets.

The higher final weight and ADG for lambs fed the protein supplement diet corresponds with their higher DMI compared to the other diets. Results concur with Obeidat et al. (2020) who reported high final body weights and ADG when feeding high protein diets compared to low protein forage diets. The high ADG observed in lambs supplemented with *A. dealbata* may be partly explained by slightly higher dietary CP and faster adaptation of animals to somewhat high dietary CT diet compared to those on the *A. mearnsii* diet. Declining weights recorded for lambs fed *A. mearnsii* up to week 6 may be reflective of strenuous adaptation to the lower CT diet. Overall, animals fed diets high in CT are likely to adapt faster than those fed diets contain less CT (Min et al., 2021). The low ADG for the control diet can be explained by the low levels of protein in the diet, which might have failed to meet the 7% CP requirements for the maintenance of the grazing dry ewes (Nicol and Brooks, 2007).

Average daily gain values (13–25 g/day) recorded in the current study fall far short of the 50 g/day minimum range required to maintain grazing lambs (Vipond et al. 2009; Roberts, 2020; 2022). This could be linked to a combination of low biomass, nutrient content, and digestibility of the rangeland forages in the current study. Average biomass values of 0.6 t/ ha estimated for the communal grazing in the study area (Matshawule, 2020) were lower than 2 t/ ha, the minimum biomass recommended to meet energy requirements for maintenance of sheep grazing dry rangelands (Roberts, 2020). Kym et al. (2018) revealed that when energy falls below maintenance for grazing sheep, protein is also utilised less efficiently.

The lowest coccidia faecal oocyst counts observed in the protein supplement diet could be attributed to the better nutritional profile resulting in stronger immune system of the lambs (Ingvartsen and Moyes, 2013; Bobeck, 2020). Inclusion of CT levels below 1.6% previously led to no effect on FEC in sheep (Hoste et al., 2006). Generally, the ability of an animal to respond to parasite infections is highly associated with nutritional status, especially protein levels (Hoste et al., 2006) and the content of CT in the diet (Mahachi et al., 2020). The inclusion of tanniferous-rich feeds with at least 2.5% CT in small ruminants’ diets has been reported to suppress parasitic infections through inhibiting nematode egg hatching and larval eclosion (Priolo et al., 2000; Hoste et al., 2006; Mahachi et al., 2020). The FEC trend reported in the current study contrasts earlier report by Cenci et al. (2007), who recorded significant effects of a tanniferous diet on FEC after the eighth week and partly related it to the adaptation of the animal to the diet. The difference with the trend presented in the current study could be largely due the low CT content of the diet as well as short duration of the current trial.

The initial low mean FAMACHA scores indicated that lambs were healthy and non-anaemic at the beginning of the trial. High mean scores at the end of the trial were a proxy of mildly anaemic and anaemic ewes which reflected a significant increase in worm burdens. Worm burdens might have presented an additional nutrient demand (Max et al., 2007) and competed for the nutrients with lambs leaving them in a negative nutrient balance. Overall, the low ADG and final body weights recorded in the current study could have been influenced by a combination of low nutrient quality of the communal rangelands and presence of gastrointestinal worms.

## Conclusions

It was concluded that supplementing low-quality communal rangelands with *A. dealbata* leaf-meal mimics commercial protein maintaining lamb growth performance compared to *A. mearnsii* leaf-meal, but both do not affect worm burdens. A follow-up study is recommended to determine the optimum inclusion levels of *A. dealbata* leaf-meal so that worm burdens are lowered while maintaining the growth performance of lambs grazing low-quality communal rangelands in the dry season.

## Data Availability

The datasets generated during and/or analysed during the current study are available from the corresponding author on reasonable request.
